# A New Multi-Modal Data Fusion Framework for Delamination Detection in Concrete Bridge Decks

**DOI:** 10.3390/s26123926

**Published:** 2026-06-20

**Authors:** Maria Rashidi, Shayan Ghazimoghadam, Vahid Mousavi, Sattar Dorafshan, Behruz Bozorg

**Affiliations:** 1 Centre for Infrastructure Engineering (CIE), Western Sydney University, Penrith, NSW 2751, Australia; m.rashidi@westernsydney.edu.au; 2 Urban Transformations Research Centre (UTRC), Western Sydney University, Parramatta, NSW 2150, Australia; b.bozorg@westernsydney.edu.au; 3 Department of Civil Engineering, Sha.C., Islamic Azad University, Shahrood 36167, Iran; shayan.ghazimoghadam@iau.ir; 4 Advanced Transportation Infrastructure Centre, University of North Dakota, Grand Forks, ND 58201, USA; sattar.dorafshan@und.edu

**Keywords:** bridge deck delamination, multi-modal data fusion, defect detection, ground-penetrating radar (GPR), infrared thermography (IRT), structural health monitoring (SHM), sensor fusion

## Abstract

Bridge decks are continuously subjected to high environmental exposure, traffic loading, and material aging, leading to progressive delamination which can negatively affect structural integrity and public safety. More specifically, subsurface delamination of concrete and corroded steel reinforcement must be repaired to keep the decks operational. Among non-destructive evaluation techniques, Ground-Penetrating Radar (GPR) and Infrared Thermography (IRT) offer complementary capabilities for detecting subsurface and near-surface defects; however, effective GPR-IRT data fusion remains challenging due to fundamental differences in sensing principles, spatial resolution and sensitivity. This study introduces a Physics-Enhanced Multi-Modal Fusion (PE-MMF) framework that integrates GPR and IRT data to improve delamination detection in reinforced concrete bridge decks. The proposed approach leverages transfer learning, cross-modal attention mechanisms, and gated fusion to enable robust learning from heterogeneous sensor inputs. Furthermore, a systematic feature selection protocol is integrated to identify physically meaningful indicators that remain consistent across different bridges, enhancing generalization capability. The framework is trained and validated using the publicly available SDNET2021 dataset, comprising co-registered GPR and IRT measurements from five in-service bridge decks with verified delamination ground truth. Results demonstrate substantial performance improvements, with average F1-score gains of up to 55% over IRT-based methods and 25% over GPR-based methods across all tested bridges. Comparative analysis against state-of-the-art methods confirmed the superior generalization capability of the proposed multi-modal approach over single-modality approaches. The findings highlight the potential of deep learning-based sensor fusion as a scalable and data-efficient decision-support tool to prioritize regions for detailed physical investigation during long-term infrastructure monitoring.

## 1. Introduction

Bridge infrastructure is a critical asset within modern transportation systems [1]. The systematic long-term monitoring of bridges is essential for preserving structural integrity and serviceability, and several studies have integrated heterogeneous monitoring data to enhance performance assessment and infrastructure-level decision-support systems, further ensuring the safety and reliability of transportation infrastructure [2,3,4]. Reinforced concrete bridge decks often deteriorate more rapidly than other bridge components due to the combined effects of traffic loading, environmental exposure, material aging and winter maintenance practices [5,6,7]. This accelerated degradation highlights the need for efficient and reliable monitoring to support condition assessment, timely intervention, and safe, cost-effective asset management [8,9].

Delamination is one of the most critical forms of deterioration affecting reinforced concrete bridge decks. While several mechanisms may initiate delamination, corrosion of reinforcing steel is the dominant cause, as the volumetric expansion of corrosion products induces internal stresses that separate concrete from the reinforcement [10,11]. If not detected and mitigated in a timely manner, delamination can propagate, leading to cracking, spalling, and a reduction in load-carrying capacity, thereby compromising both structural safety and serviceability.

Regular inspection and monitoring are essential for the early identification of both surface and subsurface defects, enabling timely intervention and preventing the progression of minor damage into serious structural deterioration. Such proactive maintenance supports sustainable infrastructure management by extending service life, reducing repair costs, and improving network reliability [12]. While traditional acoustic techniques, including hammer sounding and chain dragging, are widely used, they are subjective and often yield inconsistent results. In contrast, advanced Non-Destructive Testing (NDT) methods, such as Ground-Penetrating Radar (GPR) [13,14], impact echo [15,16], acoustic sounding [17] and Infrared Thermography (IRT) [18,19] provide more reliable assessments of internal deck conditions, allowing repeated, non-invasive evaluation and early detection of hidden defects.

Among these methods, GPR and IRT have gained increasing recognition for their ability to deliver detailed and reliable assessments of concrete conditions. GPR is widely adopted due to its rapid data collection capability, high spatial resolution and suitability for detecting concrete cover variations, deterioration zones and environments conducive to reinforcement corrosion [8,14,20]. GPR has also demonstrated capability in estimating deck thickness, reinforcement positioning, moisture ingress, and chloride contamination [13]. However, research has demonstrated that the interpretation of GPR data in reinforced concrete bridge decks is inherently challenging due to the heterogeneous nature of concrete, signal attenuation, multiple reflections, and environmental variability [21,22].

IRT is also adopted to identify material anomalies by measuring emitted thermal radiation. It detects variations in surface temperature caused by disruptions in internal heat flow, enabling the differentiation between intact and damaged regions [23]. Irregularities in thermal response patterns facilitate the identification of subsurface defects such as delamination and voids [5,24]. In recent years, Unmanned Aerial Systems (UASs) equipped with IRT sensors have demonstrated effectiveness in NDT of bridge structures, including the detection of subsurface defects, fatigue cracks in steel bridges with fracture-critical members, and corrosion in ancillary components [25]. The UAS-IRT approach enables timely, efficient, and cost-effective inspections, allowing detection of subsurface anomalies, minimizing traffic disruptions, enhancing inspector safety, and providing access to areas that are difficult or unsafe to reach using conventional methods [26,27]. However, several studies reported that while IRT is effective in locating delamination, it provides limited information regarding defect depth and is influenced by environmental conditions such as inspection timing, wind speed and ambient temperature [28,29].

The combined use of GPR and IRT represents a robust approach for concrete inspection [30]. GPR is effective in identifying subsurface anomalies and characterising internal structural conditions, whereas IRT enables rapid, non-contact detection of surface and near-surface defects. When integrated, these complementary techniques provide a more comprehensive assessment of concrete infrastructure, allowing defects to be identified across multiple depths and spatial scales [31,32].

Previous studies have demonstrated that combining GPR and IRT improves defect detection reliability by compensating for the individual limitations of each method. For example, Maser [33] showed that GPR-IRT integration is effective in detecting overlay debonding and corrosion-induced delamination in bridge decks, while Abu Dabous et al. [34] confirmed that the combined approach enhances the accuracy of delamination mapping compared to single-technique inspections. Similarly, Omar et al. [35] reported that integrating visual inspection, IRT, and GPR enables simultaneous identification of surface deterioration and subsurface corrosion-related defects, resulting in a more holistic condition assessment. Omidi [36] also combined IRT and GPR to experimentally investigate subsurface delamination in reinforced and unreinforced concrete slabs containing artificially induced defects, and demonstrated that IRT effectively detected most delamination during the cooling phase while GPR accurately identified all subsurface anomalies, including the smallest and deepest defects, confirming the complementary strengths of the two techniques for comprehensive concrete condition assessment. These findings underscore that GPR-IRT integration enhances defect-detection capabilities and enables the development of efficient, multiscale, data-rich inspection frameworks for reinforced concrete infrastructure [23,37].

However, the practical implementation of GPR-IRT fusion faces significant challenges due to differences in measurement principles, spatial resolution, environmental sensitivity, and the heterogeneous nature of the collected data [38,39]. Recent advances in deep learning (DL), particularly convolutional neural networks (CNNs), offer a promising solution by enabling automated feature extraction and classification directly from inputs, improving the potential for reliable data fusion and defect characterization [40,41,42], as demonstrated in infrastructure inspection applications such as crack detection in concrete road surfaces under varying imaging, weather, and illumination conditions using Faster R-CNN model [43]. For instance, recent research has demonstrated that CNN-based models can robustly predict rebar corrosion levels from long-term, noisy GPR B-scan data without requiring explicit environmental data [44]. Furthermore, features are scalable and comparable when developed on a validated delamination detection dataset.

Existing DL approaches for delamination detection in bridges predominantly focus on a single NDT data source or rely on laboratory specimens and numerical simulations, which do not reflect the full complexity of in-service structures and lack validation on independent, real-world datasets. Consequently, developing robust DL-based methods to translate fused GPR-IRT data into quantitative assessments of defect presence and severity remains an open research challenge.

Moreover, while some existing DL studies report exceptionally high classification accuracies, these results are often derived from datasets where training and testing samples are drawn from the same bridge deck [7,45]. Such approaches risk overfitting to bridge-specific features rather than learning the invariant physical signatures of delamination. Thus, these models often fail to generalize when applied to entirely new structures, severely limiting their practical utility. Generalization across different bridge structures remains a critical and underexplored challenge.

The Structural Defect Network (SDNET) 2021 dataset [30] provides a unique opportunity to address these gaps, offering standardized, high-quality data from five in-service bridge decks that support the training and evaluation of DL models for reliable multi-modal delamination detection. The rarity of peer-reviewed investigations on delamination detection in bridge decks using DL-based GPR-IRT data fusion highlights the originality of proposed work and the potential for further research in this area.

This study presents an initial investigation of the GPR-IRT data fusion to develop a Physics-Enhanced Multi-Modal Fusion (PE-MMF) framework for classifying bridge decks as either normal or delaminated. A multi-modal fusion model incorporating transfer learning (TL), cross-modal attention, and gated fusion mechanisms is proposed to enhance delamination detection performance. The model is trained and validated using real-world data collected from five in-service bridge decks, with delamination ground truth established through sounding and destructive verification methods. The main contributions of this study are as follows:

1. Development of a PE-MMF architecture for bridge deck delamination detection with novelty arising from the problem-driven adaptation and integration of established framework components to address cross-bridge generalization across previously unseen bridge structures.
2. Introduction of an enhanced feature extraction strategy to improve the robustness and effectiveness of multi-modal data fusion.
3. Training and validation of the proposed framework using real-world data from the publicly available SDNET2021 dataset.
4. Comparative evaluation of the proposed approach against state-of-the-art methods using the same dataset.
5. Critical analysis of the limitations of the proposed framework and its implications for model generalization and future GPR–IRT fusion research.

The remainder of this paper is organized as follows: Section 2 introduces the proposed multi-modal data fusion architecture. Section 3 details the SDNET2021 dataset. Section 4 reports real bridge case study results and provides comparative analysis with recent studies. Section 5 presents an ablation study evaluating the contribution of individual components within the PE-MMF model. Section 6 discusses the potential limitations of the proposed framework. Finally, Section 7 concludes the paper with final remarks and future research directions.

## 2. Proposed Methodology

This study proposes a PE-MMF framework designed to detect delamination in reinforced concrete bridge decks by integrating information from infrared thermography, ground-penetrating radar data, and a set of engineered features. By combining these distinct modalities, the framework aims to leverage the surface-level thermal information provided by IRT and the subsurface structural insights offered by GPR to achieve robust classification performance.

**Figure 1:**
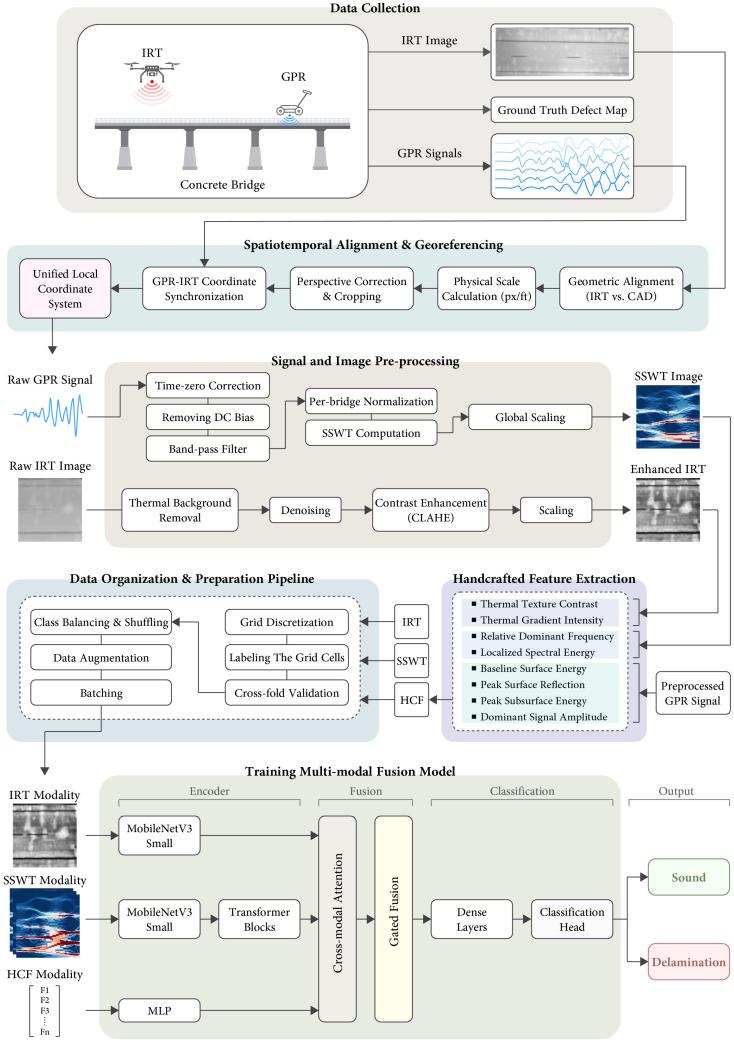
The overall workflow of the proposed PE-MMF methodology.

The overall workflow of the proposed methodology is illustrated in Figure 1. It summarizes how raw field measurements are collected, aligned within a unified coordinate system, pre-processed, and transformed into inputs suitable for model training and evaluation. To provide a clear overview, the process begins with data collection and the establishment of a unified local coordinate system to align the disparate modalities. Following alignment, raw GPR signals and IRT images undergo specific pre-processing steps to enhance signal quality and remove environmental noise. To generate training samples, the bridge decks are discretised into grids, and a rigorous labelling strategy is applied. Additionally, a set of physics-based handcrafted features (HCFs) is extracted to serve as a third modality. Finally, these inputs are processed by a multi-modal fusion model that utilizes TL, cross-modal attention, and gated fusion mechanisms to classify regions as either normal or delaminated. In this study, the term physics-enhanced reflects two aspects: (1) the HCFs are directly linked to the known physical behaviour of delamination in GPR and IRT signals, rather than being generic statistical descriptors; and (2) the feature selection protocol retains only features that remain consistently discriminative across all bridge structures, prioritizing physical stability over bridge-specific patterns. The specific procedures and algorithms implemented at each stage of this pipeline are detailed in the following subsections.

### 2.1. Data Acquisition

In this study, the non-destructive evaluation data were gathered using IRT and GPR. IRT images were captured with UAS equipped with a radiometric infrared camera flown at low altitude above the deck. The flight path ensured consistent image overlap, allowing the individual frames to be assembled into an ortho-mosaic map that represented the full deck surface. This approach provided a uniform thermal view suitable for later alignment and grid-based sampling.

GPR data were collected by moving a high-frequency antenna across the deck along predefined longitudinal and transverse scan lines. The antenna produced a series of one-dimensional radar traces that recorded subsurface reflections from rebar, voids, and potential delamination regions. For a comprehensive description of the data acquisition protocols and equipment specifications, readers are referred to the original dataset publication [5].

### 2.2. Spatiotemporal Alignment and Georeferencing

To enable the multi-modal fusion of IRT imagery and GPR signals, a precise spatial correspondence between the two modalities was required. As illustrated in the alignment module of Figure 1, a unified local coordinate system (ULCS) was established to bridge the domain gap between the GPS-tagged GPR traces and the high-resolution but non-georeferenced IRT imagery.

As raw IRT images lacked geospatial metadata, bridge design drawings (CADs) were utilized as the geometric ground truth. Deck boundaries were extracted from the CAD files, and the IRT ortho-mosaics were registered onto this reference frame. A homography transformation was computed to orthorectify the imagery, correcting perspective distortions and establishing a consistent physical scale $S_{px}$ across the bridge deck. The transformation mapping a physical coordinate (x,y) in the CAD system to a pixel coordinate (u,ν) in the IRT image is defined by the affine transformation matrix, as follows:

(1) $$\left[\begin{array}{c} u\\ \nu \\ 1 \end{array}\right]=\left[\begin{array}{ccc} s\cos\theta & -s\sin\theta & t_{x}\\ s\sin\theta & s\cos\theta & t_{y}\\ 0 & 0 & 1 \end{array}\right]\left[\begin{array}{c} x\\ y\\ 1 \end{array}\right]$$

where s represents the scale factor (pixels/ft), θ is the rotation angle required to align the bridge axis, and $\left(t_{x},t_{y}\right)$ are the translation offsets. Following this registration, GPR signal traces were mapped into the ULCS using the derived parameters. This synchronization ensured that every GPR scan was spatially indexed to its corresponding surface thermal intensity, enabling precise pixel-level fusion.

### 2.3. Signal and Image Pre-Processing

This stage prepares both sensing modalities for fusion by converting the GPR traces into time–frequency images and enhancing the IRT imagery to mitigate environmental noise, standardize inputs, and enhance defect visibility.

#### 2.3.1. GPR Signal Processing and Time–Frequency Transformation

**Figure 2:**
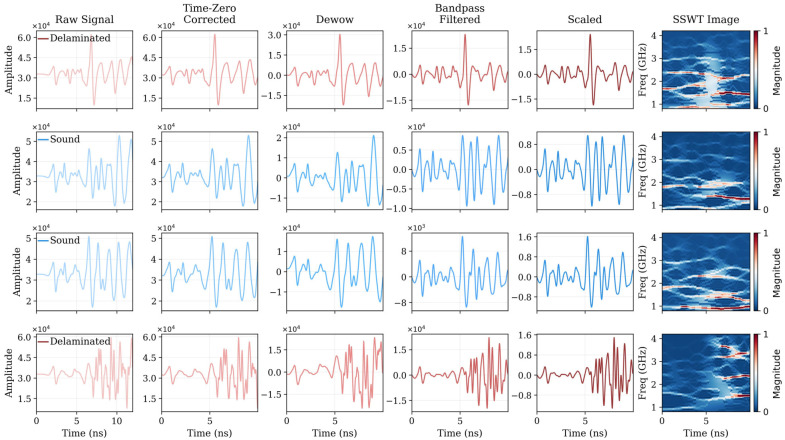
The results of transformation of a raw GPR signal into the SSWT spectrogram.

Raw GPR A-scans often contain noise, DC offsets, and temporal misalignments that can obscure subsurface features. A multi-stage processing pipeline was implemented to isolate the structural response:

1. Time-Zero Correction: To compensate for vertical bouncing of the GPR cart and variations in ground height, the direct coupling pulse (the first reflection from the surface) was aligned to a fixed time index across all traces and bridges.
2. Dewow and Filtering: Low-frequency inductive trends and DC bias were removed using a high-pass filter. Subsequently, a band-pass filter was applied to suppress high-frequency noise outside the antenna’s operating bandwidth, enhancing the signal-to-noise ratio.
3. Synchro Squeezed Wavelet Transform (SSWT): To capture the non-stationary nature of electromagnetic waves propagating through deteriorating concrete, the 1D time-domain signals were transformed into 2D time–frequency representations. Unlike the standard Continuous Wavelet Transform (CWT), which often produces blurry images due to energy spreading, SSWT concentrates this energy to create a sharper, high-resolution profile that makes subtle defect signatures easier to distinguish [7]. The CWT, which serves as the basis for this process, is defined as:

(2) $$W_{x}\left(a,b\right)=\frac{1}{\sqrt{a}}\int_{-\infty }^{\infty }x\left(t\right)\psi^{*}\left(\frac{t-b}{a}\right)dt$$

where ψ is the Morlet wavelet, a is the scale, and b is the translation. Following this transformation, the SSWT reassignment sharpens the smearing in $W_{x}\left(a,b\right)$ by mapping the energy from the scale domain a to the frequency domain ω, resulting in a high-resolution 2D image suitable for CNN-based analysis. Figure 2 illustrates the transformation of a raw GPR signal into the SSWT spectrogram. In the resulting spectrograms, sound concrete typically exhibits a uniform, low-complexity frequency distribution. In contrast, delaminated regions display chaotic patterns with concentrated high-frequency components.

#### 2.3.2. IRT Image Enhancement

Raw thermal imagery collected by UAS is frequently degraded by non-uniform heating and low thermal contrast. To address this, the IRT imagery was pre-processed to compensate for uneven illumination, suppress sensor noise, and emphasize temperature gradients related to debonding. This pipeline effectively separates local delamination anomalies from environmental artifacts. The enhancement process consisted of three key steps:

1. Thermal Background Removal: To eliminate low-frequency illumination gradients caused by solar angle and shadowing, the global background trend was estimated using a wide-kernel Gaussian filter. This trend was subtracted from the original IRT image to produce a residual image that isolates local thermal anomalies.
2. Denoising: A bilateral filter was applied to the residual image. This technique reduces high-frequency sensor noise while strictly preserving the sharp thermal edges that characterize the boundaries of delaminated areas.
3. Local Contrast Enhancement: Finally, before scaling, Contrast Limited Adaptive Histogram Equalization (CLAHE) was utilized to maximize the visibility of defects. By redistributing local intensity histograms, CLAHE ensures that subtle temperature differences indicating early-stage delamination are amplified, even within regions that were originally low-contrast.

**Figure 3:**
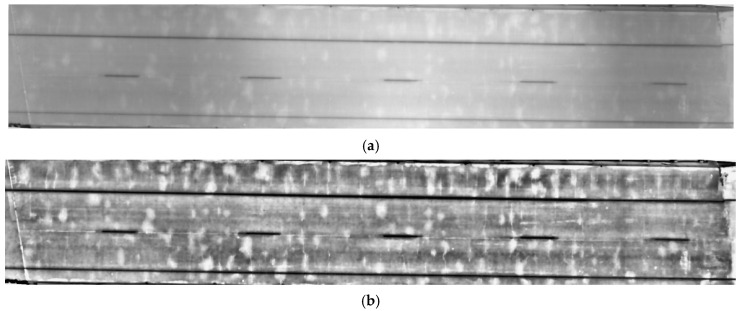
IRT image Enhancement results; (**a**) the georeferenced and cropped IRT image; (**b**) the final enhanced version.

Figure 3 presents a comparison between the georeferenced, cropped IRT image and the final enhanced version, demonstrating the improved visibility of subsurface defects after processing.

### 2.4. Handcrafted Feature (HCF) Extraction

A key component of the proposed PE-MMF framework is the incorporation of delamination-specific HCFs, which constitute the physics-enhanced element of the framework. Each HCF is directly linked to a known physical behavior of delamination. GPR-based features capture changes in signal amplitude and energy distribution caused by air voids between concrete layers. SSWT-based features track the frequency shift and resonance effects that occur when electromagnetic waves pass through deteriorated concrete. IRT-based features measure the thermal contrast and boundary sharpness that arise when subsurface delamination disrupts heat flow through the deck. This physically grounded design was important because IRT and GPR measurements can be noisy and variable, making it difficult for a learning-based model to consistently identify subtle defect-related cues without explicit domain guidance.

To determine the optimal set of HCFs, a large pool of statistical, spectral, and textural features was initially extracted from the IRT, GPR, and SSWT modalities. A systematic selection protocol was then applied to identify features that are both discriminative and robust to domain shifts. The selection process involved ranking features based on their individual separability scores, quantifying their ability to distinguish between sound (Class 0) and delaminated (Class 1) classes. Crucially, to ensure the generalizability of the proposed framework, this ranking was analysed across data from multiple bridges. Only those features that demonstrated consistent high performance across all bridges were retained. This filtering process reduced the initial high-dimensional feature set to a compact vector of the most robust domain-specific indicators. The retained features capture distinct physical phenomena associated with concrete delamination and are organized into three categories based on their source modality. Table 1 summarizes the selected HCFs along with their physical interpretation and relevance to delamination detection. These selected features form the HCF input vector, which is normalized using robust scaling statistics to ensure numerical stability during model training.

### 2.5. Data Organization and Preparation

Following the enhancement of signal and image data and the HCF extraction process, the disparate modalities were spatially associated and structured into unified samples suitable for multi-modal learning. To achieve this, a grid discretization strategy was implemented. The bridge deck surface was divided into non-overlapping grid cells (e.g., 1 × 1 ft), consistent with milling, serving as the fundamental unit for data alignment. Within each grid cell, data from all three modalities were co-registered, ensuring that every training sample contained a localized IRT context patch, a sequence of SSWT images, and the associated vector of HCFs.

However, thermal anomalies associated with delamination often span areas larger than a single grid unit, and their identification requires comparison with the surrounding sound concrete. Consequently, the IRT patches extracted for each sample were sized larger than the physical grid cell to provide essential spatial context. To ensure the model prioritizes the central grid cell, while still benefiting from this context, a spatial weighting mask was applied. This mask preserves the full intensity of the central region while attenuating the brightness of the surrounding periphery, effectively guiding the network’s attention to the specific area being labelled without losing the broader thermal perspective.

To assign a single ground-truth label to each grid cell, the spatially aligned GPR traces were utilized. The ground truth was defined entirely by physical inspections (chain dragging and milling). As provided in the SDNET2021 dataset, each GPR trace was assigned a label based on its physical location. Therefore, the traces act only as spatial carriers of the verified labels. To prevent noisy annotations near defect boundaries, a 90% purity threshold was applied. A grid cell was assigned a specific class only if at least 90% of the GPR traces within that cell shared the same label. Grid cells that did not meet this threshold were removed from the dataset. A 90% purity threshold was selected as a balance between retaining sufficient training samples and minimizing label noise near defect boundaries, which is critical for stable DL training. This step was necessary to prevent label noise caused by assigning binary labels to physically mixed cells at defect boundaries.

To evaluate the model’s generalization capability, a cross-fold validation scheme was employed using a “Leave-One-Bridge-Out” approach. In each fold, data from one bridge were held out strictly for testing, while the remaining bridges were used for training.

In real-world infrastructure assessments, collected data is frequently characterized by a significant class imbalance, where sound concrete vastly outnumbers defective regions. To prevent the model from biasing toward the majority class, class balancing and shuffling were integrated into the pipeline. During the training phase, samples were drawn dynamically from the sound and delaminated pools to ensure that every training batch maintained an equitable class distribution. Furthermore, the dataset was globally shuffled to remove any spatial or temporal correlations between consecutive samples.

To further improve robustness against environmental variability, data augmentation was applied online during data loading. This process focused specifically on the IRT modality, as thermal imagery is highly sensitive to surface conditions. Transformations including random horizontal and vertical flips, brightness adjustments, and noise injection were applied to the thermal patches to prevent overfitting.

Finally, the pipeline concluded with batching, where the processed, balanced, and augmented samples were grouped into mini batches, preparing the inputs for the multi-modal fusion model.

### 2.6. Multi-Modal Fusion Model Architecture

**Figure 4:**
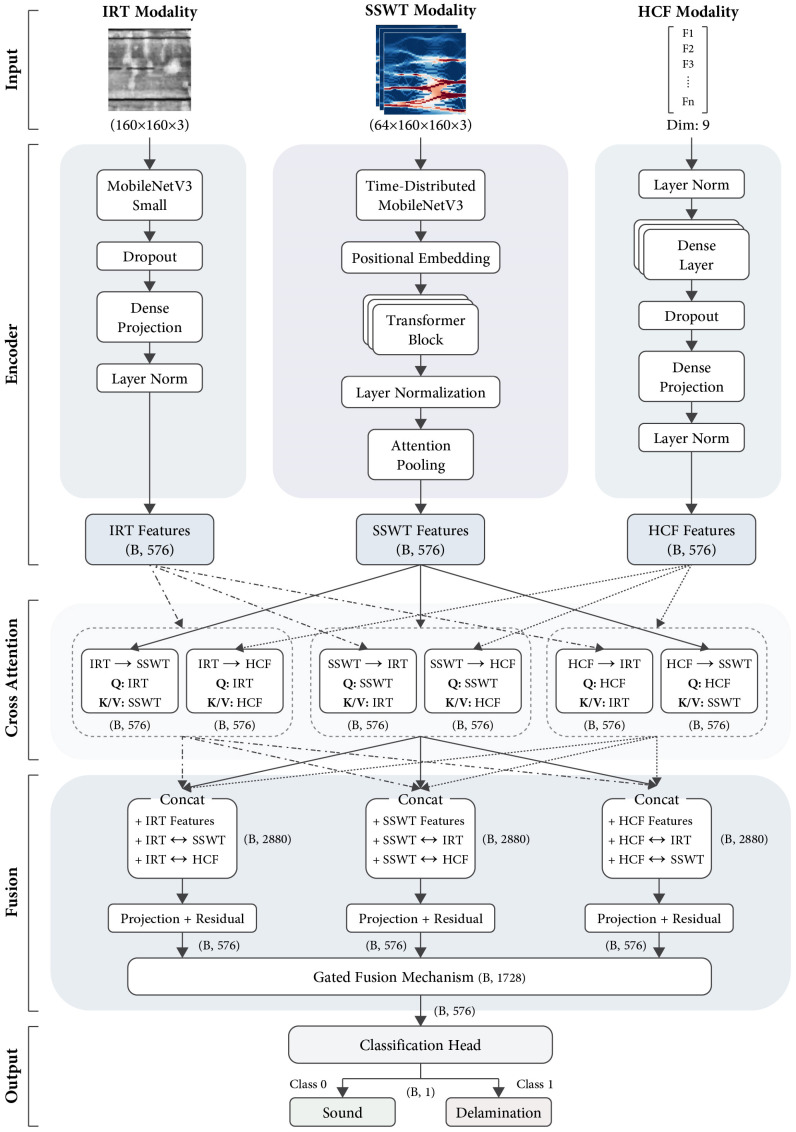
The proposed multi-modal fusion model architecture.

As illustrated in Figure 4, the proposed framework is designed to process three distinct data streams simultaneously: the IRT image, the sequence of SSWT images, and the vector of HCF. Given the limited size of the dataset, training deep convolutional networks from scratch poses a significant risk of overfitting. To address this, TL was employed by utilizing MobileNetV3-Small backbones initialized with pre-trained ImageNet weights. This specific architecture was selected for its compact size and computational efficiency, which prevents GPU memory exhaustion (OOM) and accelerates the training process while still providing robust feature extraction capabilities.

While the IRT and HCF branches rely on standard encoding, the SSWT modality requires a more complex strategy due to its sequential nature. This branch consists of 64 time–frequency images per grid cell, representing the subsurface profile. This input size was uniformly downsampled from approximately 90 raw traces recorded per grid, selected as a trade-off to optimize computational efficiency while maintaining sufficient spatial density for defect characterization. To capture the temporal relationships between these images, a Transformer block is applied after the initial feature extraction. Furthermore, a grid cell often contains a mixture of sound and delamination signals, where delamination signatures may appear in only a few specific traces or SSWT images. To address this, an attention pooling layer was incorporated at the end of the SSWT encoder. This mechanism enables the model to focus specifically on the frames exhibiting defect patterns, assigning them higher importance while filtering out the non-informative sound traces within the same cell.

To bridge the semantic gap between these distinct data types, a bidirectional cross-modal attention mechanism was implemented. This design allows every modality to query the others for relevant context; for example, the IRT branch can verify if a surface hot spot coincides with a subsurface anomaly detected by the GPR. By concatenating these attention-derived context vectors with the original features, each modality is enriched with complementary information from the other streams without losing its unique physical characteristics.

Finally, the enriched features are integrated using an adaptive gated fusion mechanism. In real-world inspections, one sensor may occasionally be compromised. For instance, IRT data might be affected by shadows, or GPR signals by electromagnetic noise. To handle this variability, the gating mechanism dynamically calculates a learned importance weight for each branch for every individual sample. This allows the model to adjust the relative contribution of each modality and reduce the influence of less informative inputs before generating the final classification probability.

### 2.7. Training

The proposed multi-modal fusion framework was implemented using TensorFlow. The network parameters were optimized using the AdamW algorithm. This optimizer was selected for its decoupled weight decay mechanism, which provides superior regularization for complex architectures involving Transformers and attention mechanisms compared to standard stochastic gradient descent. The training objective was minimized using the binary cross-entropy loss function.

To maximize the benefits of TL while mitigating the risk of overfitting, a multi-stage progressive unfreezing strategy was adopted. Initially, the pre-trained backbones were kept entirely frozen, allowing only the custom projection heads, cross-modal attention modules, and the gated fusion layer to learn from scratch. This approach prevents the large gradients generated by the randomly initialized layers from disrupting the well-formed feature representations of the pre-trained encoders. Once the new layers stabilized, the backbones were gradually unfrozen in subsequent stages, starting from the higher-level layers and progressing toward the entire network. This allowed the model to fine-tune abstract, domain-specific features before optimizing the lower-level feature extractors.

To ensure numerical stability throughout this process, differential learning rates were applied, with significantly lower rates used during the fine-tuning phases to preserve feature integrity. A dynamic learning rate scheduler was employed to monitor validation performance; the learning rate was automatically decayed whenever the improvement in the evaluation metric plateaued, allowing the model to converge to an optimal solution.

## 3. Dataset

**Figure 5:**
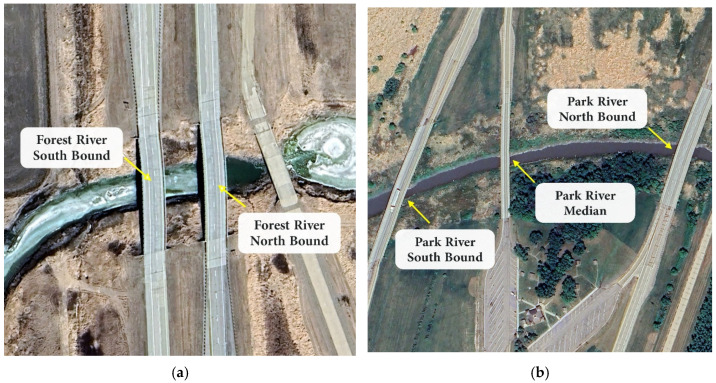
The satellite view of bridges; (**a**) the Forest River bridges and (**b**) the Park River bridges. (Source: Google Earth).

This study utilizes the SDNET2021 dataset [30], which comprises NDE data collected from five in-service reinforced concrete bridge decks located in North Dakota, USA. As illustrated in Figure 5, the study area encompasses the Forest River (North and South Bound) and Park River (North Bound, South Bound, and Median) bridges. These structures, constructed between 1971 and 1973, were subjected to varying environmental conditions and traffic loads, providing a realistic testbed for defect detection algorithms.

In this dataset, the labels were generated through a multi-stage physical validation process during scheduled deck repairs, ensuring that the ground truth reflects the actual physical condition of the concrete. The process began with the removal of the top 75 mm of the concrete deck via milling. Following milling, a physical inspection crew conducted a chain-drag survey on the exposed concrete surface to identify subsurface delamination. The boundaries of the suspected delaminated regions were physically marked on the deck and mapped using high-precision GPS. To confirm the depth and severity of the identified defects, the marked regions were subjected to progressive mechanical excavation. Regions where concrete was removed using a jackhammer down to the level just above the top rebar mat were categorized as shallow delamination (Class 1). These exposed regions were chain-dragged again to check for deeper deterioration; if further defects were detected, the concrete was excavated below the top rebar mat, establishing the boundaries for deep delamination (Class 2). Portions of the deck that did not show deterioration during the visual and physical inspections were designated as sound concrete (Class 0). AutoCAD was used to generate detailed, georeferenced survey maps of these verified physical boundaries, which served as the geometric ground truth for the NDE data.

For the purpose of this multi-modal fusion framework, specific data selection and preprocessing steps were necessary. Although Impact Echo (IE) data is available in SDNET2021, it was excluded from this study due to its extreme spatial sparsity. Furthermore, while the IRT imagery covers the entire surface of all five bridges, the GPR data collection was limited to specific scan lines and sub-sections for most decks, with the exception of the Park River Median (MD) bridge which has near-complete coverage.

To address the inherent scarcity of deep delamination samples (Class 2) and to prevent the model from learning biased representations, the classification task was simplified to a binary problem. In the SDNET2021 dataset, deep delamination accounts for only about 5.6% of the total GPR signals. Because deep learning models require sufficient sample volumes to converge, Class 1 and Class 2 were aggregated into a single “Delaminated” category, while Class 0 was retained as “Sound.” Despite this aggregation, the dataset remains naturally imbalanced, with sound concrete significantly outnumbering defective regions. This characteristic reflects the reality of field inspections and necessitates the rigorous class balancing strategies for training deep learning models.

## 4. Results and Discussion

To evaluate the effectiveness of the proposed multi-modal fusion framework, a comprehensive set of experiments was conducted on the SDNET2021 bridge deck dataset. The experiments were designed to examine whether the integration of IRT, GPR, and domain-specific HCF improves the detection of delamination across different bridge structures. The evaluation focuses on assessing both predictive performance and the model’s ability to generalize to new environments.

To ensure the reliability of the results and mimic real-world deployment scenarios, a Leave-One-Bridge-Out (LOBO) cross-validation strategy was employed. In each fold, the model was trained on four bridges and tested on the remaining unseen bridge. This approach tests the model’s ability to generalize to new environments without overfitting to specific structural configurations or ambient conditions.

### 4.1. Spatiotemporal Alignment Verification

The spatiotemporal alignment strategy detailed in Section 2.2 was validated to ensure precise geometric correspondence between the modalities. The raw IRT imagery was transformed and georeferenced to match the ULCS established by the GPR data. This process allows for the pixel-level association of surface thermal patterns with subsurface radar reflections.

**Figure 6:**
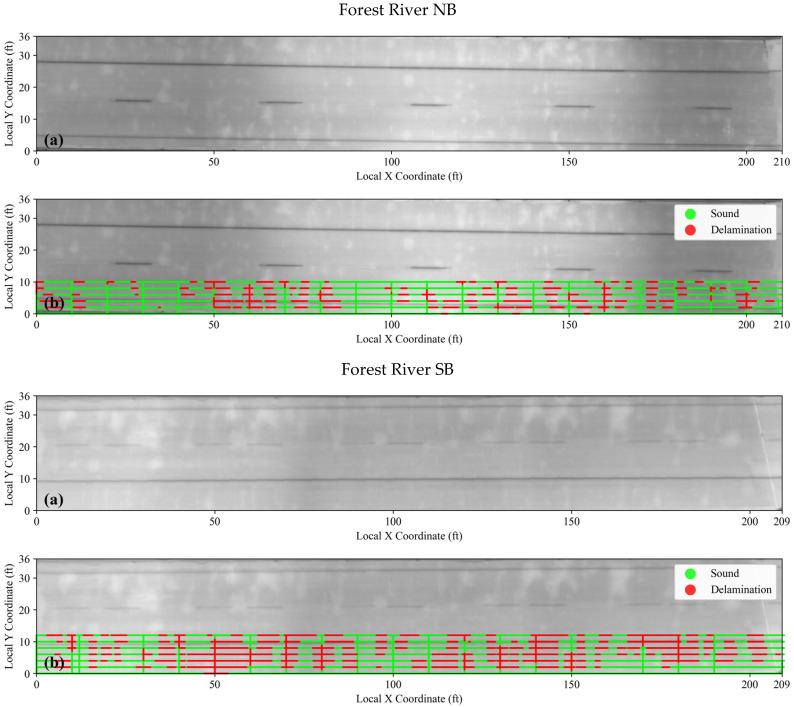

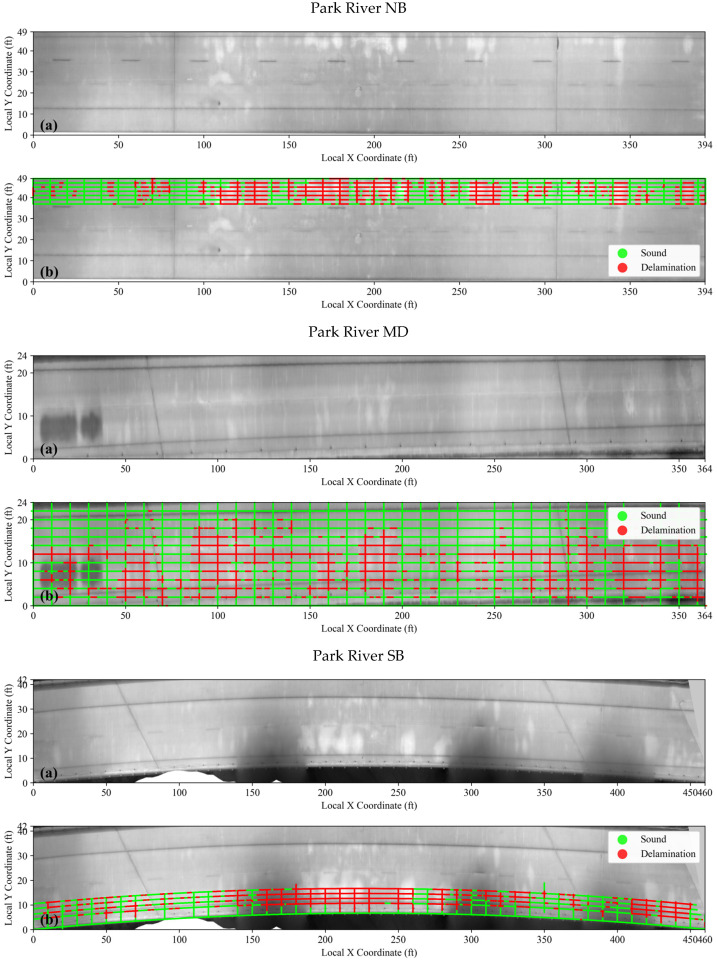
Spatial alignment validation on all five bridges. (**a**) The georeferenced and cropped IRT imagery within the local coordinate system. (**b**) The same IRT view with overlaid GPR traces, where green indicates sound concrete and red indicates delamination.

This alignment is visually demonstrated in Figure 6, using the Park River North Bound bridge as a representative example. Figure 6a presents the final georeferenced thermal map, cropped to the physical boundaries of the bridge deck. In Figure 6b, the recorded GPR scan lines are overlaid directly onto the thermal image. The traces are color-coded based on the ground truth analysis, where green represents sound concrete and red indicates delamination.

As observed in the overlay, the GPR data collection was limited and did not cover the entire surface area of the deck. Consequently, regions of the IRT image that lacked corresponding GPR coverage were excluded from the input dataset. During the grid discretization process, only grid cells containing valid co-registered data from both modalities were retained for input.

### 4.2. HCF Consistency Analysis

To ensure the multi-modal framework generalizes effectively across different bridges and environmental conditions, a feature selection protocol was implemented. As introduced in Section 2.4, a diverse set of statistical, spectral, and textural features was initially extracted from the pre-processed IRT, GPR, and SSWT data. The primary objective of incorporating these HCFs was to guide the fusion model toward the fundamental physical signatures of delamination, thereby mitigating the risk of overfitting to environmental noise or bridge-specific artifacts.

The discriminative strength of each feature was quantified using three separability metrics, including Area Under the ROC Curve (ROC-AUC), Absolute Cohen’s d, and the Kolmogorov–Smirnov (KS) statistic. For each bridge, these metrics were combined into a composite rank score that reflects how well a feature differentiates between sound and delaminated regions. This evaluation was performed independently for all five bridges to assess cross-domain consistency.

**Figure 7:**
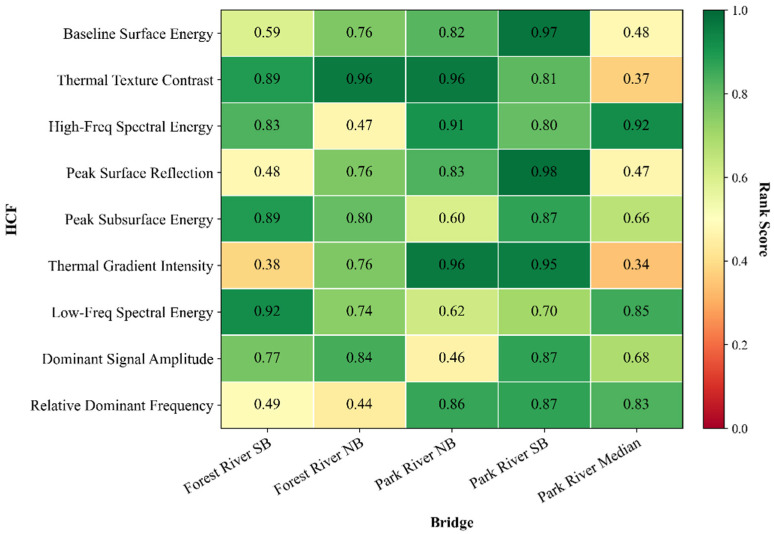
Heatmap of HCF separability rank scores across five bridges. Higher scores indicate stronger discrimination between sound and delaminated regions.

The consistency of the retained features is visualized in Figure 7. In this heatmap, higher values (indicated in green) represent stronger separability between sound and defective concrete. As observed in the figure, feature performance varies significantly across different bridges. This fluctuation is attributed to variations in deck construction, material properties, measurement quality, and environmental conditions. For instance, thermal features that perform well on the Forest River bridges may show reduced separability on the Park River MD due to different surface contrast conditions. To mitigate this issue, only the features that demonstrated consistently high rank scores across all bridges were retained.

This selection strategy prioritizes cross-bridge stability to ensure the model relies on globally valid, physically meaningful indicators of subsurface deterioration. The nine retained features, shown in Figure 7, represent those most robust to domain shifts. It is important to note that this consistency analysis was performed on the entire dataset prior to the LOBO cross-validation to establish a fixed, domain-informed feature set. Despite this global selection, the nine retained features still exhibit low separability on specific bridges, confirming that the model still faces realistic domain shifts during testing. By incorporating these consistent HCFs into the multi-modal fusion framework, the model is guided toward delamination-related signatures rather than noise or bridge-specific artifacts. This design choice improves generalization and is one of the major advantages of the proposed multi-modal approach.

### 4.3. Experimental Setup

#### 4.3.1. Dataset Composition and Input Definition

For signal and image processing, the same preprocessing pipeline was used for IRT and GPR data, as described in Section 2.3. Following the preprocessing and alignment procedures, the bridge decks were discretised into non-overlapping 1 × 1 ft grid cells, which served as the basic spatial unit for modality alignment and labelling, as described in Section 2.5. Within each grid cell, three modalities were co-registered to form a single training sample: (1) an IRT patch, (2) a sequence of 64 SSWT images, and (3) a 9-dimensional vector of selected HCF. This resolution was selected as a strategic trade-off. It is sufficiently large to capture stable GPR statistics and recognizable thermal signatures of delamination yet remains fine enough to provide the spatial localization accuracy required for practical structural assessment.

After discarding impure cells that did not meet the 90% label-purity threshold, the preprocessing pipeline produced 12,021 labelled samples across the five bridges. This step removed approximately 5 to 10 percent of the initial grid cells. Impure cells were discarded to ensure cleaner and more reliable training data. As detailed in Table 2, the data distribution varies across the five bridges, with the Park River MD bridge contributing the largest volume of samples due to its extensive GPR coverage. The aggregated dataset exhibits a natural class imbalance, consisting of 68.6% sound concrete (Class 0) and 31.4% delaminated concrete (Class 1). This imbalance confirms the necessity of the dynamic class-balancing strategy employed during training.

#### 4.3.2. Implementation Details

To address the inherent class imbalance in the dataset, a dynamic balanced sampling strategy was integrated into the data loader. During training, batches were constructed by sampling equally from the sound and delaminated classes, preventing the model from biasing toward the majority class. Furthermore, to enhance the model’s robustness against environmental variability, online data augmentation was applied, as described in Section 2.5.

The proposed multi-modal fusion framework was implemented using TensorFlow. All experiments were executed on NVIDIA A100 GPU with 40 GB of VRAM. The complete training pipeline, including the progressive unfreezing stages described in Section 2.7, required approximately 30 h to complete 250 epochs per cross-validation fold. The complete inference pipeline was evaluated on the Park River NB test set (2674 grid cells) as a representative case. The model processed each grid cell in approximately 13.0 ms, completing the full bridge assessment in approximately 34.7 s on the same GPU. At inference, the framework requires less than 10 GB of GPU memory. The training process utilized a binary cross-entropy loss function. The specific hyperparameters used for model configuration and training are summarized in Table 3.

### 4.4. Modality Contribution Analysis

**Figure 8:**
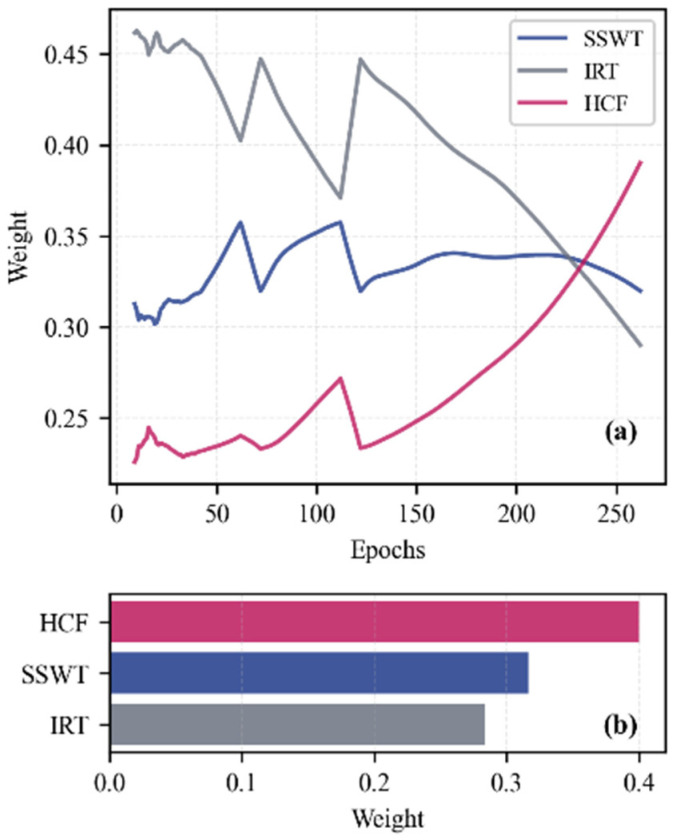
Gated fusion modality weights for a representative training fold: (**a**) weight evolution during training and (**b**) end-of-training weights.

To quantify the relative importance of each modality during the classification process, the learnable weights associated with the adaptive gated fusion mechanism (described in Section 2.6) were monitored throughout training. These weights represent the learned relative importance of each modality in the classification decision, determining how much influence each modality exerts on the final prediction. This mechanism assigns normalized weights to the IRT, SSWT, and HCF modalities, allowing the model to adjust their relative importance during training. The evolution of these weights and the final learned values are shown in Figure 8.

As shown in Figure 8a, the training trajectory is marked by distinct fluctuations around epochs 60 and 120, corresponding to the progressive unfreezing phases where the model adapts to new trainable parameters. Initially, IRT dominates the decision process, likely due to the pre-trained backbone’s rapid ability to latch onto high-contrast surface thermal patterns. Furthermore, the majority of delaminated regions were shallow where IRT is more sensitive. However, as training progresses, the IRT weight steadily declines. This decrease likely reflects the high variability of thermal imagery across bridges. The SSWT branch shows moderate and stable weighting.

In contrast, the weight assigned to HCF modality steadily increases and becomes dominant by the end of training, as shown in Figure 8b. This trend indicates that the selected HCFs provide the most stable and discriminative cues across all bridges, capturing underlying physical signatures of delamination that are less affected by domain shifts and noise. Overall, these results highlight the benefit of combining sensor modalities with well-designed, physically meaningful HCFs within a gated fusion mechanism.

It is noted that Figure 8 presents the weight trajectory from one training fold (Park River NB as the held-out test bridge) to illustrate the general learning behavior, and weight dynamics can vary across folds due to differences in bridge characteristics and data. Furthermore, the model checkpoint used for evaluation in each fold was selected based on the best validation F1-score, so the terminal weights in Figure 8b do not necessarily correspond to the gating configuration at the saved checkpoint.

### 4.5. Quantitative Performance Comparison with Baselines

To benchmark the performance of the proposed PE-MMF framework, the results were compared against two recent state-of-the-art studies utilizing the SDNET2021 dataset. These studies represent the model generalization capability of single-modality DL approaches:

- IRT-UNet: A semantic segmentation approach proposed by Ichi and Dorafshan [5], utilizing a U-Net architecture trained exclusively on IRT images.
- GPR-1D-CNN: A signal classification approach proposed by Elseicy et al. [45], utilizing a 1D-CNN applied to GPR A-scans.

It is important to note that this evaluation represents an indirect pipeline-level comparison rather than a strictly controlled benchmark. Since the baseline methods were not retrained using the same grid-cell definition, purity rule, preprocessing strategy, binary label structure, and LOBO folds, they rely on their originally published dataset partitioning and preprocessing strategies. Applying the specific preprocessing protocol of the PE-MMF framework to these baselines would fundamentally alter their respective methodologies. Therefore, this comparison evaluates the complete end-to-end pipelines against the shared challenge of cross-bridge generalization using the SDNET2021 dataset.

The F1-score was selected as the primary evaluation metric due to the class imbalance between sound and delaminated concrete. Table 4 summarizes the comparative results across all five bridge decks. As presented in the table, the PE-MMF framework consistently outperforms both single-modality baselines. When compared to the IRT-UNet baseline, the fusion model demonstrates a massive performance uplift, particularly on the Park River NB bridge, where the F1-score increased from 0.362 to 0.667 (+84.3%).

The proposed framework also yields significant gains over the stronger GPR-1D-CNN baseline. On the Park River MD and SB bridges, the fusion model improved upon the GPR results by 32.8% and 45.7%, respectively.

It is important to contextualize these results within the inherent complexity of the SDNET2021 dataset. These five bridge decks exhibit significant heterogeneity regarding rebar distribution, concrete cover thickness, and historical maintenance interventions. This structural variability creates a challenging domain shift for testing the model generalization. The fact that the single-modality baselines struggle significantly on specific bridges (e.g., Park River SB) suggests that they overfit to the specific features of the training bridges and fail to generalize to new structural conditions. The superior performance of the PE-MMF framework indicates that fusing complementary modalities, including thermal surface data with radar depth data, allows the model to learn more invariant defect representations that remain robust even across differing operational and environmental conditions.

**Figure 9:**
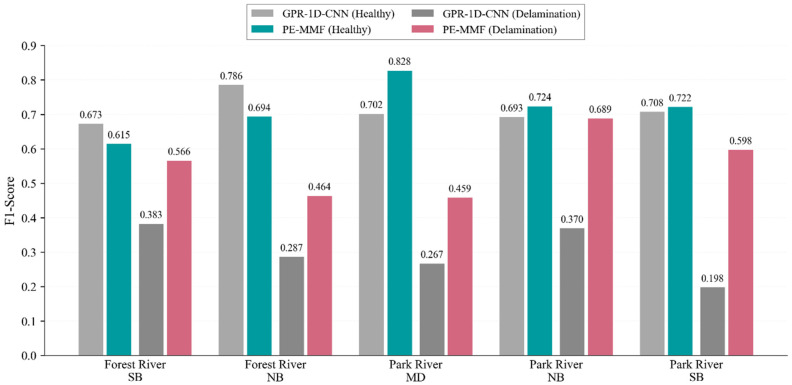
Per-class F1-score comparison between the GPR-1D-CNN and the PE-MMF framework across five bridges.

To further examine model behavior under class imbalance, a per-class F1-score comparison with the GPR-1D-CNN baseline is presented in Figure 9. For the delamination class, which is the minority and primary target in inspection tasks, PE-MMF yields substantial increases in F1-score across all bridges, ranging from 47% to over 200% improvement. For example, on Park River SB, the delamination F1-score increases from 0.198 (GPR-1D-CNN) to 0.598 using PE-MMF. These improvements indicate that the proposed framework is particularly effective at identifying defective regions, even when the available training samples are limited and highly variable.

Overall, the results demonstrate that the integration of multiple sensing modalities, combined with physics-enhanced HCFs and adaptive fusion, substantially improves delamination detection performance compared to systems relying on a single sensing source. This improvement is particularly noteworthy given the inherent limitations of the SDNET dataset, including variability in environmental conditions during data acquisition, differences in material properties, and inconsistent delamination severity across bridges [30]. While models trained and tested on data from the same bridge can achieve high accuracy (up to approximately 80%) [7], performance typically declines when applied to different bridges due to distributional shifts and bridge-specific characteristics. Although it is well recognized that many deep learning models do not consistently achieve uniform accuracy across different bridge evaluation scenarios [46], the proposed model demonstrates strong generalization capability, achieving robust cross-bridge performance despite the dataset’s variability and inherent limitations.

### 4.6. Qualitative Detection and Localization Results

**Figure 10:**
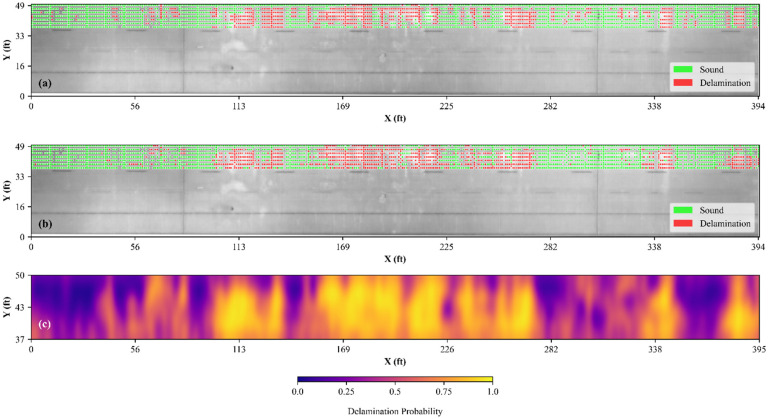
Delamination detection results for Park River NB bridge (unseen test set): (**a**) ground truth labels; (**b**) model predictions; and (**c**) predicted delamination probability heatmap.

To complement the quantitative metrics, the spatial distribution of the detection results is visualized in Figure 10. These results represent the Park River NB bridge, which served as a completely unseen test case during the cross-validation process. The predictions are mapped onto the georeferenced IRT imagery using the ULCS.

Figure 10a displays the ground truth labels, where red indicates delamination and green indicates sound concrete. The binary predictions generated by the PE-MMF model are shown in Figure 10b. A visual comparison shows a strong correspondence between the predicted defects and the ground truth. The model successfully localizes the primary clusters of delamination, particularly in the heavily deteriorated sections of the bridge deck.

In addition to binary classification, the framework outputs a continuous probability score, visualized as a heatmap in Figure 10c. Brighter colours indicate a high probability of delamination, while darker hues represent sound concrete.

Consequently, these qualitative results show that PE-MMF produces coherent and physically meaningful delamination maps on a bridge not seen during training. The use of local coordinates also supports practical inspection workflows by enabling direct localization of predicted defects on the bridge surface.

By producing these detailed probability heatmaps, the framework enables deployment as a decision-support tool during field inspections. Instead of performing comprehensive manual surveys of entire bridge decks, inspectors can use the PE-MMF model’s predictions to prioritize regions requiring detailed physical investigation. This creates a human-in-the-loop workflow where automated screening reduces inspection time and labour while the probability heatmaps guide inspectors to efficiently locate potential delamination zones for verification.

## 5. Ablation Study on PE-MMF Model

To assess the contribution of each component within the proposed PE-MMF framework, an ablation study was conducted on the Park River NB bridge, which served as a representative unseen test case. Six configurations were evaluated, ranging from single-modality models to complete fusion architecture. The settings included IRT only, SSWT only, HCF only, the combination of IRT and SSWT, the combination of all three modalities without the cross-modal attention and gated fusion mechanisms, and the full PE-MMF model.

Five metrics were reported in total. Precision measures the proportion of predicted delamination cells that are truly delaminated, while recall measures the proportion of actual delaminated cells that the model correctly identifies. ROC-AUC describes the model’s ability to separate sound and delaminated cells across all decision thresholds, and PR-AUC reflects the same separability but focuses on the minority delamination class, which makes it more informative under the class imbalance present in this study.

The results of all six configurations are summarized in Table 5. Among the single-modality results, IRT provided the strongest standalone performance. This outcome was expected because of the shallow delamination regions, for which surface thermal contrast is informative. However, the moderate precision indicates that IRT alone was still affected by thermal artifacts and bridge-specific surface variations. In other words, some sound cells were likely classified as delaminated when their thermal response resembled a defect pattern.

The SSWT-only model produced the weakest results. Its ROC-AUC was only slightly above random separation, and its recall was low. This suggests that the SSWT representation, when used alone as a high-dimensional image sequence, did not provide stable defect cues for this unseen bridge. Several factors may explain this behavior. First, the SSWT input is derived from local GPR traces, and delamination signatures may appear only in a small portion of the 64-frame sequence within a grid cell. Second, the time–frequency images can contain redundant or noisy texture patterns that are difficult to learn from the limited number of bridge samples. Third, the MobileNetV3 backbone was pre-trained on natural images, not GPR time–frequency representations, which may limit its ability to extract meaningful SSWT-specific features without larger training data. Fourth, without the attention pooling and the contextual guidance provided by the other modalities, the branch struggles to isolate these sparse cues from the dominant sound response. Therefore, the poor SSWT-only result does not necessarily indicate that GPR information is unhelpful. Rather, it suggests that SSWT images alone may not be the most robust way to represent GPR information under the present dataset size and cross-bridge testing condition.

The HCF-only model showed a different behavior. Although its overall F1-score was lower than that of IRT, its recall remained relatively high. This indicates that the selected HCFs captured useful physical signatures associated with delamination, but they were not sufficiently discriminative when used without spatial image context. The low precision suggests that HCFs alone tended to over-identify delamination. Nevertheless, the HCF-only result confirms that these features contain complementary information and can support the fusion model.

When IRT and SSWT were combined, the recall increased substantially and reached the highest value among all configurations. This result is important from an inspection perspective because missed delamination zones have a higher safety cost than false alarms. The high recall indicates that combining surface thermal information with GPR-derived time–frequency information helped the model identify a larger portion of the actual delaminated cells. However, this improvement was accompanied by lower precision. Therefore, the IRT plus SSWT model was more sensitive to delamination, but it also produced more false positives. Such a model may be useful for conservative screening, but it is less balanced for practical decision support.

Adding HCFs to IRT and SSWT without attention improved the overall balance of the model. Precision and PR-AUC increased compared with the IRT plus SSWT configuration, while recall decreased. This shows that the HCFs helped suppress some false alarms by adding compact physical descriptors to the learned image features. However, without cross-modal attention and gated fusion, all active modalities were combined by simple concatenation. As a result, the model could use additional information, but it could not explicitly adjust the relative contribution of each modality for each sample.

The full PE-MMF model achieved the best overall performance. It produced the highest F1-score, precision, ROC-AUC, and PR-AUC, while maintaining a strong recall. Compared with the model without attention, the full architecture improved both precision and recall, indicating that the cross-modal attention and gated fusion mechanisms contributed positively. This result suggests that the model benefited from allowing each modality to exchange information before final classification. It also suggests that the gated fusion mechanism helped reduce the influence of noisy or less reliable modalities on individual samples.

Overall, the ablation study confirms that no single modality was sufficient to provide robust delamination detection on the unseen bridge. IRT was the strongest standalone modality, SSWT alone was not reliable in this setting, and HCFs provided useful but incomplete physical information. The best performance was obtained when the three modalities were integrated through the full PE-MMF architecture.

## 6. Potential Limitations

Despite the superior performance of the PE-MMF framework compared to single-modality baselines, several limitations should be acknowledged. First, the generalization capability of the model is constrained by the size and heterogeneity of the available data. While the SDNET2021 dataset provides high-quality measurements, the variability between the five bridge decks ranging from differences in cover thickness and rebar distribution to varying maintenance histories creates a significant domain shift. As observed in the results, the model performance fluctuates depending on which bridge is held out for testing, with F1-scores ranging from 0.579 to 0.667 across the five test bridges (Table 4), highlighting the sensitivity of the model to bridge-specific characteristics. To achieve a truly invariant model capable of handling any reinforced concrete deck, a larger, more diverse dataset encompassing a wider range of structural designs and environmental conditions is required.

Second, the reliability of the ground truth labels presents an inherent challenge. The labels were derived from chain dragging and destructive verification; however, perfect pixel-level alignment between these physical measurements and the NDE data is difficult to achieve. Potential inaccuracies or noise in the ground truth can confuse the model during training, particularly at the boundaries of delaminated regions. The 90% label-purity threshold applied during grid cell labelling was intended to reduce this effect, but boundary ambiguity cannot be fully eliminated.

Third, partial GPR coverage is a core limitation for full-deck mapping. While UAS-based IRT provides comprehensive coverage of the entire deck surface, GPR data collection is often limited to specific longitudinal scan lines or subsections, with only the Park River MD bridge achieving near-complete GPR coverage. Because the PE-MMF model requires inputs from both modalities, this spatial mismatch necessitates the exclusion of valid thermal imagery where corresponding radar data existed. Consequently, the model could not fully utilize the thermal data.

Fourth, while the HCFs were selected based on established physical principles of delamination, the consistency analysis was performed at the full-dataset level prior to the LOBO validation. Although no model parameters were trained or tuned on the test bridge, this global feature-selection introduces a minor potential risk of information leakage during the model-design stage. Future work could perform feature selection independently within the LOBO training fold to ensure complete methodological isolation between training and testing data.

Fifth, the ablation study revealed limitations in how individual modalities are represented, particularly the GPR data. The poor standalone performance of the SSWT modality suggests that extracting non-stationary electromagnetic wave signatures using 2D time–frequency images and backbones pre-trained on natural images is challenging with a limited dataset. Without the spatial context of IRT or the physical anchoring of HCFs, the sparse defect cues within the 64-frame SSWT sequences are easily overshadowed by the dominant sound concrete response. Future works on this framework could explore alternative preprocessing methods, 1D sequence models, or domain-specific pre-training for GPR signals.

Finally, although the proposed framework improved the F1-score on unseen bridges, this metric indicates that false positives and false negatives still occur. For example, on the Park River NB bridge, the full PE-MMF model achieved a precision of 0.622 and recall of 0.719, meaning approximately 28% of actual delaminated cells were missed and some sound cells were incorrectly flagged. Further refinement is necessary to reach the reliability levels required for automated decision-making without human oversight. Given that delamination is a form of structural deterioration that may influence local mechanical behaviour and deformation response [47], human oversight remains essential. Nevertheless, the generated probability maps may support increasing levels of automation in inspection planning and asset management workflows as model performance improves.

## 7. Conclusions

This research presented a Physics-Enhanced Multi-Modal Fusion (PE-MMF) framework for delamination detection in reinforced concrete bridge decks through the fusion of ground-penetrating radar (GPR) and infrared thermography (IRT) data. By leveraging the complementary sensing characteristics of these two non-destructive evaluation techniques, the proposed approach addressed key limitations of single-modality methods and demonstrated improved robustness for real-world bridge assessment. The framework was developed and evaluated using the publicly available SDNET2021 dataset, which contains co-registered multi-modal measurements from five in-service bridge decks with verified delamination ground truth, enabling transparent benchmarking and reproducible evaluation.

The proposed framework achieved substantial improvements over state-of-the-art single-modality methods, demonstrating improvements of 55% and 25% in average F1-score compared to IRT-based and GPR-based methods, respectively. These improvements were particularly pronounced for the delamination class, where the fusion strategy proved especially effective. The Leave-One-Bridge-Out (LOBO) cross-validation results confirm that the framework effectively generalizes to unseen bridge structures, a critical requirement for practical deployment.

Analysis of the adaptive gating mechanism revealed that handcrafted features (HCFs) provide the most stable discriminative cues across different bridges, highlighting the value of incorporating domain knowledge into data-driven models. The systematic feature selection protocol, which retained only features demonstrating consistent performance across multiple structures, proved essential for achieving robust generalization.

Evaluation using metrics including precision, recall, ROC-AUC, and PR-AUC indicated that the full PE-MMF architecture improves the balance between false alarms and missed defects. Additionally, the ablation study suggested that a single modality might not provide adequate reliability for unseen bridge structures. Cross-modal attention and gated fusion mechanisms were proven effective for suppressing noise.

The proposed fusion architecture effectively learned discriminative features from heterogeneous sensor inputs, enabling reliable delamination classification while mitigating challenges associated with environmental variability, material heterogeneity, and sensor-specific noise.

The identified limitations, including dataset size, variability across bridge designs, partial GPR coverage, and uncertainty in ground truth labels, indicate that further improvements are required before fully automated deployment. Nevertheless, the proposed framework represents a meaningful step toward reliable multi-modal bridge inspection in real-world conditions. A practical advantage of this framework is its potential to integrate into standard bridge management workflows. Rather than replacing current methods, it functions as a decision-support tool that translates multi-modal sensor data into spatial probability maps for delamination detection. Bridge managers can use these results to more efficiently prioritize inspection resources, locate priority zones, and schedule maintenance interventions. However, the limitations discussed earlier indicate that human verification remains necessary before making final repair decisions.

Future work will focus on several directions: first, extending the framework to quantify delamination severity, moving beyond binary classification to continuous damage assessment; second, incorporating temporal inspection sequences to track damage progression and predict future deterioration patterns; third, investigating more powerful transfer learning (TL) architectures, such as ResNet or EfficientNet, which may further improve feature extraction and detection performance compared to the lightweight MobileNetV3 backbone used in this study; fourth, developing advanced domain adaptation strategies to enhance generalization across diverse bridge inventories with varying structural configurations, materials, and environmental conditions; and fifth, integrating the framework with digital twin platforms and bridge asset management systems to support data-driven decision-making and long-term infrastructure monitoring.

## Figures and Tables

**Table 1:** Selected HCFs and their physical significance.

Modality	Feature Name	Description
GPR	RMS Energy (Surface vs. Rebar)	Quantifies energy distribution at different depths; delamination attenuates signals from rebar level
GPR	Primary Reflection Amplitude	Maximum reflection strength at reinforcement depth; indicates dielectric contrast from air voids
SSWT	Relative Dominant Frequency	Tracks spectral downshift caused by deteriorated concrete acting as low-pass filter
SSWT	Localized Spectral Energy	Detects resonance effects characteristic of subsurface voids
IRT	GLCM Contrast	Measures thermal intensity variations highlighting delamination hot spots
IRT	Gradient Magnitude Mean	Identifies sharp thermal boundaries at defect edges

**Table 2:** Distribution of valid samples across the five bridge decks after preprocessing.

Bridge Name	Total Samples	Contribution (%)
Forest River NB	1187	9.9%
Forest River SB	1346	11.2%
Park River NB	2674	22.2%
Park River MD	4413	36.7%
Park River SB	2401	20.0%
Total	12,021	100%

**Table 3:** Summary of model hyperparameters and training configuration.

Parameter	Value/Description
Input resolution (IRT/SSWT)	160 × 160 pixels
Batch size	32
Optimizer	AdamW
Learning rate (Phase 1)	1 × 10^−4^
Learning rate (Phase 2)	5 × 10^−5^
Learning rate (Phase 3)	5 × 10^−7^
Transformer blocks	3
Attention heads	4

**Table 4:** Comparison of F1-scores between the proposed PE-MMF framework and single-modality baselines, including relative performance improvements (Impr.) and overall average scores across five bridge decks.

Bridge	PE-MMF	IRT-UNet	GPR-1D-CNN	Impr. vs. IRT	Impr. vs. GPR
Forest River SB	0.590	0.42	0.528	+40.5%	+11.7%
Forest River NB	0.579	0.427	0.536	+35.6%	+8.0%
Park River MD	0.644	0.427	0.485	+50.8%	+32.8%
Park River NB	0.667	0.362	0.531	+84.3%	+25.6%
Park River SB	0.660	0.401	0.453	+64.6%	+45.7%
Average	0.628	0.407	0.507	+54.3%	+23.9%

**Table 5:** Ablation study results comparing single-modality baselines and fusion configurations on the Park River NB bridge.

Category	Ablation Setting	F1	Precision	Recall	ROC-AUC	PR-AUC
Single	IRT only	0.576	0.515	0.653	0.745	0.536
	SSWT only	0.312	0.329	0.296	0.532	0.326
	HCF only	0.476	0.377	0.645	0.616	0.381
Fusion	IRT + SSWT	0.604	0.467	0.854	0.788	0.569
	IRT + SSWT + HCF (without attention)	0.617	0.552	0.699	0.772	0.602
	Full PE-MMF	0.667	0.622	0.719	0.813	0.627

## Data Availability

The dataset used in this study is the publicly available Structural Defect Network (SDNET) 2021 dataset. The code utilised for implementation is available upon request from the Corresponding Author.

## Abbreviations

The following abbreviations are used in this manuscript:

CNN	Convolutional Neural Network
DL	Deep Learning
GPR	Ground-Penetrating Radar
HCF	Handcrafted Features
IRT	Infrared Thermography
LOBO	Leave-One-Bridge-Out
NB	North Bound
PE-MMF	Physics-Enhanced Multi-Modal Fusion
SB	South Bound
SSWT	Synchro Squeezed Wavelet Transform
TL	Transfer Learning
UAS	Unmanned Aerial Systems
ULCS	Unified Local Coordinate System
